# Discordance in self-report and observation data on mistreatment of women by providers during childbirth in Uttar Pradesh, India

**DOI:** 10.1186/s12978-017-0409-z

**Published:** 2017-11-15

**Authors:** Arnab Dey, Holly Baker Shakya, Dharmendra Chandurkar, Sanjiv Kumar, Arup Kumar Das, John Anthony, Mrunal Shetye, Suneeta Krishnan, Jay G. Silverman, Anita Raj

**Affiliations:** 1 Sambodhi Research and Communications Pvt. Ltd, C-126, Sector -2, Noida, Uttar Pradesh - 201301 India; 2 Center on Gender Equity and Health, Division of Global Public Health, University of California, San Diego School of Medicine, La Jolla, CA USA; 3 India Health Action Trust, No. 13, 1st Floor. 4th Cross, N S Iyengar Street, Sheshadripuram, Bangalore, Karnataka - 560020 India; 4 Bill and Melinda Gates Foundation, 5th Floor, Capital Court, Olof Palme Marg, Munirka, New Delhi, India

**Keywords:** Maternal health, Mistreatment, Childbirth, Abuse, Discrimination, Discordance, Observation, Self-report, India

## Abstract

**Background:**

The study aims to assess the discordance between self-reported and observed measures of mistreatment of women during childbirth in public health facilities in Uttar Pradesh, India, as well as correlates of these measures and their discordance.

**Methods:**

Cross sectional data were collected through direct observation of deliveries and follow-up interviews with women (*n* = 875) delivering in 81 public health facilities in Uttar Pradesh. Participants were surveyed on demographics, mistreatment during childbirth, and maternal and newborn complications. Provider characteristics (training, age) were obtained through interviews with providers, and observation data were obtained from checklists completed by trained nurse investigators to document quality of care at delivery. Mistreatment was assessed via self-report and observed measures which included 17 and 6 items respectively. Cohen’s kappas assessed concordance between the 6 items common in the self-report and observed measures. Regression models assessed associations between characteristics of women and providers for each outcome.

**Results:**

Most participants (77.3%) self-reported mistreatment in at least 1 of the 17-item measure. For the 6 items included in both self-report and observations, 9.1% of women self-reported mistreatment, whereas observers reported 22.4% of women being mistreated. Cohen’s kappas indicated mostly fair to moderate concordance. Regression analyses found that multiparous birth (AOR = 1.50, 95% CI = 1.06–2.13), post-partum maternal complications (AOR = 2.0, 95% CI = 1.34–3.06); new-born complications (AOR = 2.6, 95% CI = 1. 96–4.03) and not having an Skilled Birth Attendant (SBA) trained provider (AOR = 1.47, 95% CI = 1.05–2.04) were associated with increased risk for mistreatment as measured by self-report. In contrast, only provider characteristics like older provider (AOR = 1.03, 95% CI = 1.02–1.05) and provider not trained in SBA (AOR = 1.44, 95% CI = 1.02–2.02) were associated with mistreatment as measured through observations. Younger age at marriage (AOR = 0.86, 95% CI = 0.78–0.95) and provider characteristics (older provider AOR = 1.05, 95% CI = 1.01–1.09; provider not trained in SBA AOR = 0.96, 95% CI = 0.92–0.99) were associated with discordance (based on mistreatment reported by observer but not by women).

**Conclusion:**

Provider mistreatment during childbirth is prevalent in Uttar Pradesh and may be under-reported by women, particularly when they are younger or when providers are older or less trained. The findings warrant programmatic action as well as more research to better understand the context and drivers of both behavior and reporting.

**Trial registration:**

CTRI/2015/09/006219. Registered 28 September 2015.

## Plain English summary

Despite concerted global efforts to reduce mortality of women during childbirth, maternal mortality continues to be a significant cause of death among women worldwide. One of the important component of the efforts to decrease maternal mortality is to improve the quality of care received by women during delivery. While provision of quality care during delivery comprises of a number of clinical protocols, respectful care during delivery is also a major determinant of quality and has been found to be associated with critical maternal and child health outcomes.

Attention to the issue of respectful care during childbirth has increased over the past decade both from a public health and human rights perspective. While there is a growing body of literature on the subject, the field of measurement of respectful care is somewhat nascent. One measurement debate has been on whether mistreatment of women during delivery should be assessed via self-report by women or direct observation of deliveries. While both the methods have their strengths and limitations, a deeper understanding of those would help in improving the evidence and its reliability.

In this study, we assess provider mistreatment of women during childbirth via self-reports and observations and examine the concordance between these two measures. We examine characteristics of women and providers associated with each method and their discordance to provide greater insight into the nature of these measures and their potential biases. The study focuses on women delivering in public health clinics in Uttar Pradesh, the most populous state in India.

## Background

Dignified respectful healthcare for women during pregnancy and childbirth is a human right, and attention to this issue has increased over the past decade, more broadly from a human rights perspective, and more specifically from a public health lens, as mistreatment by a provider during childbirth can be associated with numerous health complications for both the mother and the child [1–5].

While there are concerted global efforts to reduce complications and mortality of women during childbirth, particularly in low resource countries, maternal mortality continues to be a significant cause of death among women worldwide [6]. An important component of many efforts to decrease maternal mortality include programs designed to increase access to quality antenatal and facility delivery services. This programmatic priority raises the issue of how quality is defined, how it can be measured, as well as the degree to which lack of quality serves as an impediment to women’s decision to utilize available care. A component of quality care that is receiving attention amongst researchers and clinicians is the issue of mistreatment of women by health care providers during childbirth. A growing body of literature suggests that fear of such mistreatment is a key impediment to timely acquisition of care and use of institutional facilities for childbirth, particularly among less educated and poor women, and is associated with poor birth outcomes for both mother and child [7–14]. Such mistreatment can include a broad array of provide behaviors, from neglectful or non-consensual care to verbal or physical abuse against a woman during childbirth [14].

Despite a growing body of evidence on the adverse effects of mistreatment during birth, quality of care in terms of respectful and compassionate treatment of women continues to receive little attention in programming efforts, likely in part because its definition and measurement remains in dispute [3, 15]. A recent comprehensive systematic review of 65 qualitative and quantitative studies on the topic documents the following major types of mistreatment by providers: direct abuse (physical, sexual or verbal), discrimination, failure to meet professional standards of care (non-consensual or non-confidential care, neglect or abandonment, and inadequate or poor quality medical resources), and non-supportive care [16]. However, authors of this review highlighted that in practice quantitative studies on the topic held “inconsistent identification criteria and operational definitions” [16]. This lack of a standard measure may partially account for the highly-varied prevalence of mistreatment reported (12–98%) across different populations and national contexts [3, 4]. While research in this area is growing, and efforts are being made to assess the prevalence of the problem, the best ways to measure mistreatment are still uncertain. One debate has been on whether self-report or direct observation would be better and more valid. Lack of clarity on measurement makes it difficult to both evaluate the full extent of the occurrence of mistreatment, as well to evaluate the factors, both determining and consequential of, its occurrence [1, 16–19].

More insight into the limitations and strengths of different ways to measure mistreatment is important, both from the perspective of collecting evidence that adds to our understanding of the scale of the problem, and from the perspective of defining mistreatment during childbirth and refining measurement tools that reflect a more precise definition. In this study, we assess mistreatment via self-reports and observations during childbirth and examine the concordance between these two sets of measures. We examine characteristics of women and providers associated with each type of mistreatment measure and their discordance to provide greater insight into the nature of these measures and their potential biases. The study focuses on women delivering in public health clinics in Uttar Pradesh, India, the largest and most populous state in a nation that accounts for 18% of all births and 15% of all maternal deaths annually [20, 21].

## Methods

Data analyzed for this study was obtained from a broader evaluation of a nurse-mentoring intervention focused on improving the quality of care in public health facilities in Uttar Pradesh; the design is described elsewhere in Raj et al. [14]. The broader evaluation involved a four-armed quasi-experimental design, and the intervention did not include components focused on reducing provider mistreatment during delivery. The analyses for this study was only post-test in nature and intervention arms were treated as covariates in the analysis to adjust for any unmeasured intervention effects.

As a part of the broader evaluation, Direct Observation of Deliveries (DODs) were conducted in Public Health facilities in Uttar Pradesh between April to August 2016 to assess the effect of the nurse-mentoring intervention on adherence to clinical protocols during delivery. A separate follow-up survey was undertaken between April and September 2016 to follow-up with the same women whose deliveries were observed within 2–4 weeks of delivery. Items used for analysis in the study include a subset of the items used in the DOD and follow-up survey.

Participants were married women aged 15–49 years who had delivered at the selected public health facilities. A total of 1047 participants were recruited for observation of deliveries study across 81 public health facilities in Uttar Pradesh. Of the women whose deliveries were observed, 83.5% were interviewed at their home within 2–4 weeks in the follow-up study (*n* = 875).

Delivery observations were undertaken by female nurses, trained on delivery care, survey research and data collection. Follow-up interviews with participants were undertaken by female research staff, trained in data collection methods, the health system context in the state, and on critical maternal and child health outcomes. Special focus was given to explaining individual survey items to the research staff and on issues of sensitivity and privacy during data collection.

For direct observation of deliveries, the Medical Officer In-Charge (MoIC) of the selected health facilities were informed about the purpose of the study, and women were informed about observers’ purpose in observing delivery care. Observations were done after obtaining written consent from the survey participants as well as health care providers. All observations started from the point a woman was admitted to the labor room and continued until she left the labor room. Observers were trained to observe the practices of delivery nurses, and note adherence to clinical protocols during initial assessments, across the various stages of delivery, and during labor monitoring. Six additional items were included on mistreatment during birth. Nurse investigators undertaking the observations were deployed in shifts over a period of 4–5 days in a single health facility to cover both day and night deliveries. All women admitted to the labor room at the sampled facilities for delivery during survey period were recruited for the study. Characteristics of health care providers providing delivery care to the women were also captured during the survey. The nurse investigators identified and coded all the health care providers present in the labor room during delivery. For each of the procedures observed during delivery, the code of the provider performing the procedure was noted by the nurse investigators. The nurse investigators then interviewed all the providers identified during the delivery observations using a structured schedule.

In case of the follow-up survey, women were informed about the study and written informed consent was obtained prior to the interviews. After obtaining consent from the participants, the interviews were conducted in a private setting. Participants in the follow-up survey were asked about their socio-demographic profile, their birth experiences at the clinic, health and services received during their antenatal, perinatal, and postnatal periods. The follow-up interview took about 60 min to complete. All data collection was done using handheld mobile devices which included no identifiable information of the participants. Data was uploaded weekly into a password protected file for data management and analysis. No monetary incentive was provided to the participants for their participation in either of the studies.

This work was conducted in partnership with the National Health Mission (NHM) of Uttar Pradesh. Institutional review board (IRB) approval for this study was granted from Public Health Service- Ethical Review Board (PHS-ERB) and from the Health Ministry Screening Committee (HMSC) facilitated by Indian Council for Medical Research (ICMR). IRB review and approval for the current analyses was obtained from University of California, San Diego.

### Measures

The primary dependent variable of interest was provider *mistreatment* of women during childbirth; this was collected via both self-report from the participants as well as via observations of deliveries. For the self-report data, we used the 8-item mistreatment measure from a prior iteration of this study in which we found the measure to have good internal reliability with a Cronbach’s alpha of 0.70 [14], but added 20 new items informed by Bohren et al. [16] on verbal abuse, lack of informed consent, lack of supportive care, lack of privacy, and facility culture. The new set of items were tested for internal consistency and 11 items that showed poor internal consistency were dropped from the scale. Dropped items included those on supportive care from provider and lack of privacy during delivery; of note, we also dropped supportive care items from our prior measure due to their poor internal consistency with the mistreatment measure as a whole [14]. Cronbach’s alpha for the final 17 item self-report measure was .72, indicating adequate internal reliability The theoretical constructs for the final 17 items included physical abuse, verbal abuse, emotional abuse, neglect and abandonment, lack of transparency in care, lack of informed consent and stigma and discrimination. (See Table 1 for the items). All of the participants were interviewed on all the 17 items during the follow-up survey.

**Table 1:** Any instances of mistreatment of women by health care providers during childbirth, reported by women delivering at 81 public health facilities in Uttar Pradesh, India (*N* = 875)

Instance of any form of mistreatment during delivery^1^	Self-reported mistreatment during childbirth (all items) (# of items = 17) % (n)	Self-reported abuse during childbirth (items matched with those in observations) (# of items = 6) % (n)	Observed mistreatment in childbirth (# of items = 6) % (n)
Any form of mistreatment – Yes	77.3 (676)	9.1 (80)	22.4
Cronbach-alpha	0.72	0.64	0.47
Individual items			
Beaten / slapped by health care provider	0.9 (8)	0.9 (8)	3.7 (32)
Provider forcefully pushed abdomen during delivery	1.7 (15)	1.7 (15)	11.4 (100)
Provider applied force to pull baby	1.5 (13)	1.5 (13)	7.9 (69)
Provider used bad/abusive language	2.6 (23)	2.6 (23)	3 (26)
Provider threatened to slap client	2.2 (19)	2.2 (19)	3.1 (27)
Client faced problem due to unavailability of provider during delivery	5.1 (45)	5.1 (45)	3.2 (28)
Client disrespected during stay at facility	1.8 (16)	–	–
Client not provided complete information on the delivery procedures	47.8 (418)	–	–
Client not provide information on problem you might face after delivery	4.6 (40)	–	–
Provider did not answer client’s questions	29.4 (257)	–	–
Provider did not tell client about her health	43.5 (381)	–	–
Provider did not tell client about her baby’s health	38.9 (340)	–	–
Provider did not advice client on avoiding illness after delivery	55.8 (488)	–	–
Provider did not take client’s consent before conducting the delivery procedures	27 (236)	–	–
Client was denied specific things by the provider	8.6 (75)	–	–
Client was treated differently based on her caste	0.6 (5)	–	–
Client was discriminated during her stay at facility	1.4 (12)	–	–

^1^Mistreatment of women by provider during childbirth was defined based on categories taken from (Bohren et al., [16])

Of the 17 items on mistreatment in the final self-report measure, six items on mistreatment that could be observed during the period of observation were also included in the delivery observation checklist, conducted by nurse investigators as described above. These six items were used to create a measure of mistreatment based on direct observation. The theoretical constructs for the six items on mistreatment included physical abuse, verbal abuse and neglect and abandonment.

Survey data from participants were also used to capture socio-demographic characteristics and pregnancy/delivery experiences of participants; these items were adapted from the National Family Health Survey (NFHS), India’s Demographic and Health Survey (DHS) [22]. Socio-demographic items were women’s age and age at marriage of women, literacy status, caste and religion. Literacy status was assessed based on the woman’s ability to read a sentence and to write her name. We assessed household wealth using a composite measure of a wealth index constructed from individual items on household assets and amenities. To create wealth categories, we ran a principal component analysis on the individual items to generate a wealth index score which was divided into equal quintiles for analysis. This approach was based on the wealth index construction from the NFHS [22, 23]. We also included items on parity and, for the index pregnancy/childbirth, intrapartum and post-partum care, multiparous birth, low birth weight of the infant, duration of stay at facility after delivery, and maternal and newborn health complications. Maternal health complications were assessed for the pregnancy, delivery, and post-partum period separately. Maternal health complications included excessive bleeding post-delivery, high grade fever, convulsions, loss of consciousness, abdominal pain, and vaginal discharge. Newborn complications were also assessed as a part of the survey and included problems like prematurity, yellowing of skin, chest in-drawing, loss of interest in breastfeeding, weakness, drowsiness etc.

In addition, we included measures on provider characteristics, captured through structured interviews with the specific providers providing delivery care to women. These items included provider age, years of experience, and whether the provider was trained in the Government recommended Skilled Birth Attendant (SBA) module [24].

### Data analysis

Discordance between self-report and observed items were assessed using Cohen’s Kappa scores [25, 26]. The level of concordance was categorized into five categories based on the values of Cohen’s Kappa viz. Poor (κK < 0.2), Fair (κK = 0.21–0.40), Moderate (κK = 0.41–0.60), Good (κK = 0.61–0.80) and Very good (κK = 0.81–1.0) [25, 26]. To assess characteristics of women and provider associated with mistreatment, we used three outcome variables: 1) any form of self-reported mistreatment, 2) any form of observed mistreatment, and 3) mistreatment reported via both assessments. Chi-square analyses and t-tests were conducted to determine bivariate associations between socio-demographic indicators (age, literacy, caste, household wealth, religion), reproductive health related factors (parity of participants, complications during delivery, and post-partum or new-born complications), intervention arm, and provider characteristics (age, experience, and training) with each of the three mistreatment outcomes. We then ran unadjusted and adjusted logistic regression models to assess the associations between the independent variables and provider mistreatment based on self-reports and observations. We constructed parsimonious models [22, 27] to ensure that we did not over-adjust in our analyses. In our final models, we included only those covariates that were associated with the outcome at *p* < 0.2 or if they altered the effect size of the independent variables by more than 10%.

We also created an additional outcome variable to measure discordance between the self-report and observed mistreatment measures; this variable was categorized as: cases of concordance, cases when observers reported mistreatment and women did not, and cases when women reported mistreatment while the observer did not. We identified very few cases of non-concordance when women reported mistreatment and observers did not (*n* = 33) and dropped those from consideration for the analysis. This produced a dichotomized variable: with cases with concordance between self-report and observed mistreatment vs those where observers reported mistreatment but women did not (lower self-reported mistreatment). We again ran unadjusted and adjusted logistic regression models to assess the associations between the independent variables and our non-concordance outcome, using parsimonious adjusted models as described above [22, 27]. All analyses were conducted using STATA 13 software (StataCorp, USA).

## Results

Participants’ age ranged from 17 to 49 years (mean age = 26.8, Std. dev. = 5.1) (See Table 3). The mean age at marriage was 19.5 years (Std. dev. = 1.9). More than half of participants (52.5%) were literate i.e. they could read and write. Most participants (84.1%) were Hindu, and 88.1% belonged to either scheduled caste/scheduled tribe (SC/ST) or the Other Backward Classes (OBC), the most socially vulnerable caste groups.

Health providers at delivery were predominantly staff nurses (88.6%) followed by Auxiliary Nurse Midwives (ANMs) (9.4%). 94% of the deliveries were conducted by 1 Staff-Nurse or ANMs supported by a unskilled birth attendant. The average age of providers was 36 years (std. dev. = 11.1) and the average number of years working in the field was 10 (std. dev = 10.0). Almost half of the providers (47.4%) had received the Government of India recommended Skilled Birth Attendant training.

Majority of women (77.3%) self-reported mistreatment by their provider, most commonly in the areas of non-consensual care or inadequate information provisions regarding treatment or care for them or their child. (See Table 1). When we limit the self-report measure to the 6 items included in the observation measure, only 9.1% reported mistreatment by their provider; this is notably lower than that reported by observers on these same items (22.4%), which focused on provider abuse and harsh delivery practices, as well as non-presence of the provider.

A review of self-reported and observer-reported mistreatment by item demonstrates higher reporting by observers as compared to reports by women for all abuse and harsh delivery-focused items but not for the non-presence of provider item. Provider forcefully pushing the woman’s abdomen was observed in 11.4% of deliveries and self-reported by only 1.7% of the participants. Disparities were also found in other items of physical abuse including women beaten/slapped by provider (observation = 3.7%; self-report = 0.9%) and provider applying force to pull the baby during delivery (observation = 7.9%; self-report = 1.5%). Cohen’s Kappa scores were also used to assess concordance between self-report and observation data and reported moderate level of concordance on items related to verbal abuse and threats from the provider; concordance on items related to physical abuse, harsh delivery practices, and non-presence of the provider ranged from fair to poor (See Table 2).

**Table 2:** Cohen’s Kappa scores of concordances between instances of observed and reported mistreatment reported by women delivering at 81 public health facilities in Uttar Pradesh, India (*N* = 875)

Individual items	Cohen’s Kappa scores	Quality of agreement^a^
Beaten / slapped by health care provider	0.39	Fair
Provider forcefully pushed abdomen during delivery	0.17	Poor
Provider applied force to pull baby	0.25	Fair
Provider used bad/abusive language	0.52	Moderate
Provider threatened to slap client	0.46	Moderate
Client faced problem due to unavailability of provider	0.33	Fair

^a^Quality of agreement between observed and recorded observations based on categories taken from Kwiecien et al.

Bivariate analyses (chi-squares and t-tests) indicate that the same variables did not correlate with both self-reported and observed mistreatment from providers (See Table 3). Regression analyses found that births that were multiparous (AOR = 1.50, 95% CI = 1.06–2.13), resulting in post-partum maternal health complications (AOR = 2.0, 95% CI = 1.34–3.06) or new-born complications (AOR = 2.6, 95% CI = 1. 96–4.03), and attended by providers who had not received SBA training (AOR = 1.47, 95% CI = 1.05–2.04) were associated with self-reported mistreatment. Women delivering in non-intervention facilities were also more likely to report mistreatment (See Table 4). In contrast, provider and facility characteristics were associated with observed mistreatment; specifically, these were cases with older providers (AOR = 1.03, 95% CI = 1.02–1.05) and non-SBA trained providers (AOR = 1.44, 95% CI = 1.02–2.02) (See Table 5). Insignificant trend associations with this outcome were also seen for longer stay at the facility and older age of mother (See Table 5).

**Table 3:** Sociodemographic characteristics, birth experiences, and perinatal health of women delivering at 81 public health facilities in Uttar Pradesh, India, for total sample (N = 875) by observed and self-reported Mistreatment of Woman by Provider During Childbirth

Variables		Self-reported mistreatment during childbirth (all items) (# of items = 17)		Self-reported abuse during childbirth (items matched with those in observations) (# of items = 6)		Observed mistreatment in childbirth (# of items = 6)	
	Total Sample (*N* = 875)	Mistreatment During Childbirth (*n* = 676)	*p*-value*	Mistreatment During Childbirth (*n* = 80)	*p*-value*	Mistreatment During Childbirth (*n* = 196)	*p*-value*
	% (n)	% (n)		% (n)		% (n)	
Sociodemographics							
Age of respondents							
Mean	26.8	26.8	0.75	28.1	0.015	26.9	0.72
Std. dev.	5.1	5.1		7.5		5.4	
Age at Marriage							
Mean	19.5	19.4	0.24	19.7	0.23	19.2	0.03
Std. dev.	1.9	1.8		2.1		1.9	
Literacy							
Illiterate	47.5 (416)	79.5 (331)	0.12	8.9 (37)	0.81	22.8 (95)	0.77
Literate	52.5 (459)	75.2 (345)		9.4 (43)		22.0 (101)	
Religion							
Hindu	84.1 (736)	77.7 (572)	0.46	9.5 (70)	0.39	22.4 (165)	0.98
Muslim	15.9 (139)	74.8 (104)		7.2 (10)		22.3 (31)	
Caste							
SC/ST/OBC	88.1 (771)	78.5 (605)	0.02	9 (69)	0.59	22.2 (171)	0.67
Other	11.9 (104)	68.3 (71)		10.6 (11)		24.0 (25)	
Wealth Index							
Lowest	19.7 (172)	77.3 (133)	0.59	12.2 (21)	0.34	23.3 (40)	0.77
Low	18.7 (164)	74.4 (122)		11 (18)		20.1 (33)	
Medium	19.1 (167)	78.4 (131)		6.6 (11)		24 (40)	
High	20.5 (179)	81 (145)		8.4 (15)		24.6 (44)	
Highest	22.1 (193)	75.1 (145)		7.8 (15)		20.2 (39)	
Parity (number of births)							
Single	32.5 (284)	73.6 (209)	0.07	9.5 (27)	0.8	21.8 (62)	0.78
Multiple	67.5 (591)	79.0 (467)		9 (53)		22.7 (134)	
Study design							
Intervention Condition			<0.001				
Nurse-mentored	51.3 (449)	70.2 (315)		10.5 (47)	0.16	22.5 (101)	0.95
Non-nurse mentored	48.7 (426)	84.7 (361)		7.8 (33)		22.3 (95)	
Stay at facility for 48 h post delivery	23.5 (206)	77.7 (160)	0.87	4.4 (9)	0.007	16.0 (33)	0.012
Any complications at delivery	40.1 (351)	80.3 (282)	0.08	12 (42)	0.02	23.9 (84)	0.37
Any complications Postpartum	28.6 (250)	86 (215)	<0.001	13.2 (33)	0.01	20.4 (51)	0.37
Any new-born complications	27.4 (240)	87.5 (210)	<0.001	14.6 (35)	0.001	25 (60)	0.26
Low birth weight newborn (<2500 g)	11.1 (97)	78.4 (76)	0.79	6.2 (6)	0.28	19.6 (19)	0.48
Age of provider							
Mean	36.3	36.2	0.57	37.9	0.18	39.7	<0.001
Std. Dev.	11.1	11.1		12.9		12.3	
Years of experience							
Mean	10.2	10.0	0.26	12.1	0.08	12.6	<0.001
Std. Dev.	10.0	9.9		11.1		11.0	
SBA (Skill Birth Attendant) trained	47.4 (415)	74.0 (307)	0.03	7.5 (31)	0.10	21.0 (87)	0.33

*p-values assess differences between groups who experienced and did not experience mistreatment during childbirth on the given variable, based on chi-square analyses for categorical variables and t-tests for continuous variables

**Table 4:** Logistic regression analyses to assess associations between equity factors, socio-economic predictors and self-reported mistreatment delivery among women delivering at 81 public health facilities in Uttar Pradesh, India (*N* = 875)

	Self-reported mistreatment during childbirth (all items) (# of items = 17)	
	Crude OR (95% CI)	Adjusted OR (95% CI)
Caste		
SC/ST/OBC	1.69 (1.08–2.65)	1.6 (0.99–2.56)
Others	Ref	Ref
Parity		
Single	Ref	Ref
Multiple	1.35 (0.97–1.87)	1.50 (1.06–2.13)
Intervention arms		
Nurse-mentored facilities	Ref	Ref
Non-nurse mentored facilities	2.36 (1.69–3.3)	2.5 (1.78–3.56)
Post-partum complications		
No	Ref	Ref
Yes	2.2 (1.5–3.3)	2.0 (1.34–3.06)
New-born complications		
No	Ref	Ref
Yes	2.5 (1.7–3.9)	2.6 (1.69–4.03)
SBA trained provider		
No	1.42 (1.03–1.96)	1.47 (1.05–2.04)
Yes	Ref	Ref

**Table 5:** Logistic regression analyses to assess associations between equity factors, socio-economic predictors and observed mistreatment delivery among women delivering at 81 public health facilities in Uttar Pradesh, India (N = 875)

	Observed mistreatment during childbirth (# of items = 6)	
	Crude OR (95% CI)	Adjusted OR (95% CI)
Age at marriage	0.91 (0.83–0.99)	0.93 (0.84–1.01)
Age of provider	1.03 (1.02–1.05)	1.03 (1.02–1.05)
SBA trained provider		
No	1.17 (0.85–1.61)	1.44 (1.02–2.02)
Yes	Ref	Ref
Stay at facility for 48 h post delivery		
No	1.69 (1.11–2.55)	1.46 (0.96–2.23)
Yes	Ref	Ref

Adjusted multivariate analysis were also conducted to predict discordance in mistreatment when observer reported mistreatment and women did not. Analyses indicated that this was more likely when women were younger at the time of marriage, (AOR = 0.86, 95% CI = 0.78–0.95), and when the delivery was conducted by older providers (AOR = 1.05, 95% CI = 1.01–1.09). The likelihood decreased with each year of experience of the provider (AOR = 0.96, 95% CI = 0.92–0.99) (See Table 6).

**Table 6:** Logistic regression analyses to assess associations between equity factors, socio-economic predictors and non-concordance in reporting mistreatment by women delivering at 81 public health facilities in Uttar Pradesh, India (*N* = 875)

	Observer reporting mistreatment when patients did not 17.7% (149)	
	Crude OR (95% CI)	Adjusted OR (95% CI)
Age at marriage	0.86 (0.78–0.95)	0.86 (0.78–0.95)
Age of provider	1.01 (1.0–1.03)	1.05 (1.01–1.09)
Years of experience of providers	1.0 (0.99–1.03)	0.96 (0.92–0.99)

## Discussion

In this study, we used unique data from a quality of care study for deliveries conducted in rural Uttar Pradesh India that included both observational and self-reported measures of mistreatment during childbirth. We were able to assess the prevalence of and the concordance between self-reported and observed mistreatment during delivery, as well as the associations between sociodemographic, childbirth complications, and provider characteristics with these mistreatment outcomes. Our findings provide an important contribution to the emerging field of study regarding respectful treatment of women during childbirth. In our prior work, one out of five women reported being mistreated by the provider during childbirth. The difference in the prevalence of reported mistreatment is attributable to the inclusion of additional items in the measure for this paper. The additional items included in the current study were mostly specific to lack of transparency in care and lack of consent. Some of these items were reported to have very high incident rates, which also led to the substantial difference in the prevalence reported in our previous work. The measure for mistreatment in our prior work had 8 items of self-reported mistreatment as compared to 17 items used in this paper. The prevalence of self-reported mistreatment based on the 17 item scale we see in this study is also much higher than the prevalence of Disrespect and Abuse reported in the 2015 Abuya et al. study in Kenya, which reported a 20% prevalence of disrespect and abuse based on 9 items around the manifestations of physical abuse, non-consented care, non-confidential care, non-dignified care, discrimination, abandonment and detention in facilities. Expanding on our prior work, we see that observers of childbirth are more likely to report mistreatment of the women than are women themselves. These findings, which attempt to triangulate self-report and observed data on this issue, suggest that, although there is likely to be a bias towards underreporting by women, mistreatment during childbirth should be a major concern in the public health system.

Because measurement of mistreatment by providers during childbirth is not yet standardized [1, 2, 14, 16], the validity of self-reported measures of abuse and disrespect during childbirth is still uncertain. The three measures of mistreatment studied in this paper viz. 17 item self-report, 6 item self-report and 6 item observation had Cronbach alpha’s of 0.72, 0.64 and 0.47 respectively. While the self-reported measures show good internal reliability, the reliability of the observed mistreatment is relatively low. Future research on mistreatment can include more items to the observations to test the internal reliability of observed mistreatment. Not only did we find higher mistreatment reported through observation relative to self-report, but the two measures showed poor concordance with each other. We also found that there was a considerable difference in the incidence of reported disrespect and abuse depending on the scale. Overall the observed measures indicated much higher rates of mistreatment compared to the self-report measures, particularly for occasions when the birth attendant pushed on the woman’s abdomen or applied force to pull the baby. This suggests that there may be under reporting of mistreatment by women, and interpretation of women’s self-reports should be considered in that light. These results are consistent with Blanc’s findings that women tended to report the quality of the facility in a more positive light than what was reported by observers [28]. It is important to note, however, that while the observers were trained in delivery care, survey research, and data collection, it is possible that being primed to look for poor quality of care, observers may be over-reporting mistreatment as well. It is also important to note that women and observers had different vantage points. For example, women might not have noticed that the provider applied force to push her abdomen or pull out the baby, which might be evident from the observer’s vantage point. Conversely, women might have reported facing problems due to the unavailability of providers during labor, which may not be evident to observers. The discordance in these measures are reflected in the low kappa scores for these indicators. To understand these dynamics more deeply, further research should consider including multiple observers, and video recording of births for expert assessment, to test inter-rater reliability. Further research should also consider discussing video recordings with women to discuss their interpretation of treatment received during delivery after the event – as opposed to relying on their memory during childbirth. Qualitative data may also be useful to help contextualize the quantitative reports.

Women who self-reported mistreatment were more likely to be from disadvantaged castes, and to be those who have had more children. While these factors can make a woman more vulnerable to abuse, given the discrepancy between the reported and observed experiences, it is also likely that there is some under-reporting taking place in the self-reports by women. From this perspective women who had already given birth several times were also potentially more experienced with the process, and so had previous experiences to draw on regarding what kind of care to expect. It may be that those women were more likely to report because of an increased awareness that the treatment they received was not optimal [29]. Nevertheless, women who self-reported mistreatment were also more likely to have given birth in a facility without a nurse mentor (non-intervention), and to have a provider that had not received SBA training, both attributes that would be beyond the knowledge of most birthing mothers, and that suggests that these self-reports of mistreatment may actually be the result of a lower standard of care in these facilities.

Women who reported mistreatment were more likely to report maternal and new-born complications. This is consistent with our previous work on the topic which showed a strong association between reported mistreatment and complications [14]. This is an important finding for several reasons. First because reduction in maternal and new-born complication is a high priority goal for programmatic efforts to improve maternity care, any findings that point to factors associated with those outcomes should be carefully considered, whether causal or not. Second, we know that maternal stress during labor can cause the labor to slow or stall, increasing risk of complications [4]. If mistreatment by a provider is shown to cause maternal complications, the reduction of such treatment would be an important focal point for interventions attempting to improve maternal care outcomes, with the added benefit that the improved treatment increases the dignity of the experience for women. With our current analyses, we cannot demonstrate causality. It is possible that women who experience complications are more likely to have an overall negative perception of the birth itself, which may predispose them to report mistreatment and abuse. Or it may be that those who are comfortable reporting complications are also more comfortable reporting maltreatment and abuse. Lastly of course, it is possible that mistreatment increases the risk of complications. The fact that we did not find this association in the analysis of observed mistreatment makes this last possibility less certain. Further research is needed to understand this association.

Consistent with what we found with the self-reported mistreatment, observed mistreatment was significantly associated with the birth attendant being untrained. The fact that we found this result with both the self-reported and observed measures suggests that provider training may be an important mechanism to improve the treatment of women during childbirth. Previous research has shown that trained providers have slightly higher levels of competence in managing complications [30], however our study is the first that we know of to show that trained providers might treat birthing women in a more respectful way. Observers were also more likely to report mistreatment if the provider was older. This may be because older providers are less up to date on current quality of care practices, and may simply treat birthing mothers more harshly. If this is the case, then why did mothers themselves not report this? In India, respect for elders is a strong social norm, and women are socialized to defer to and often accept harsh treatment from older women, particularly mother in laws [31]. It is possible then that the observers picked up on a tendency for older women to be less respectful to young birthing mothers but that those mothers did not interpret this behavior in the same way.

The dynamics of deference to elders and expectation and acceptance of harsh treatment by older women being the normative way may explain why women who were married younger than age 18 were more likely to have experienced maltreatment according to observers, but not according to their own self-report. Those married younger than age 18 are more likely to come from families that are more traditional, including adherence to very strong social hierarchies in which people of lower status, particularly very young married adolescents, receive harsh treatment in their daily lives [32]. These women may be less likely to perceive mistreatment in this context, because the treatment they received is consistent with the treatment they have received throughout their married lives. We see in fact, that the main predictors of discordance between the self-report and the observed, are these very same factors. The older the provider, and the younger the age at which the woman was married, the more likely it is that the observer noted mistreatment while the birthing mother herself did not. These findings are an important clue into the deeply engrained social norms around hierarchy and social status in which maltreatment is likely to occur. They also highlight the possibility that the discordance between self-report and observation may in fact be largely driven by under-reporting by women in the self-reported measures. Interventions to address the phenomenon of mistreatment by providers during childbirth will need to carefully address these social dynamics.

### Limitations

This study has limitations. First, the findings from this study are limited to the setting of public health facilities in Uttar Pradesh – a state with a disproportionately high number of deliveries within public health facilities [33]. While we explored the associations between mistreatment and the characteristics of the women as well as those of the providers, we could not include the delivery load borne by providers as a factor in the analysis. Further research studies are needed to explore such associations. Similar studies are also needed to understand the prevalence and nature of mistreatment for women delivering at home or private health facilities – issues that have remained largely unexplored so far.

Second, although our study had a comprehensive list of items to measure self-reported mistreatment, the number of items that were included in the observation was substantially less. This likely contributed to the far stronger internal reliability for self-reported mistreatment relative to that of the observed mistreatment measure. We also found that the associations between mistreatment and the individual and provider level predictors varied between the self-reported and observed measures. These results might also be affected by the lower number of items used in the observed mistreatment measure. In addition, the study did not include providers’ report of their behavior with women, limiting our ability to triangulate the findings. Further research is thus needed to include a comprehensive set of items captured through observations so that the observed and self-reported measures are comparable. Third, some of the self-reported and observed measures also varied based of the vantage point of women and observers, an issue which we could not address in this study. We were also unable to assess inter-rater reliability in this study. Although, all observers were trained in delivery care, research methods and data collection protocols, and stringent measures were taken to ensure data quality, we cannot ignore the possibility that observers were over-reporting mistreatment. We also recognize that there is a chance that women’s self-report on mistreatment may encapsulate their entire birth experience and be affected by poor health outcomes in spite of being asked about specific provider behavior. Such issues in self-report can be addressed by having multiple points of enquiry in the survey tools to cross check responses from women. Further research in this area should include multiple observers, or explore other methods to assess inter-rater reliability.

Assessment of mistreatment with women shortly after childbirth may also affect our findings. The timing of reports seems to play a significant role in the prevalence of disrespect and abuse reported. For instance, two separate studies conducted in Tanzania (one in a rural area, one in an urban area) found a vast increase in the prevalence of disrespect and abuse reported when interviews were conducted within the communities several weeks after the births compared to those conducted directly post-partum in the facility [34, 35]. The authors speculated that women were potentially hesitant to report abuse conducted within the facility while they were still in its care. Further, directly postpartum, women have not had the time to recover from the birth itself and process the experience, and hence are less likely to interpret events as having been abusive. In both studies, the authors also found that the women’s reports of satisfaction with care were lower in the community surveys, consistent with a time dependent post birth emotional processing experience that may be necessary to recognize and report abuse. The Sando study additionally noted that direct observers of some births reported aspects of disrespect, specifically lack of consent, lack of privacy, and non-dignified care, that were not reported by mothers potentially because such behaviors were considered normal in their communities [34].

Finally, because this is an observational study, we cannot establish causality. The relationship between mistreatment and the characteristics of women and providers suggest a causal relationship. However, we need longitudinal studies to understand the directionality of the associations. Further research on alternate methods of interviewing and observing combined with qualitative discussions with women, providers as well as observers will provide more insights into their perspectives and help in understanding the context and mechanisms of provider mistreatment as identified in the current study.

## Conclusions

Despite these limitations, this study provides important insights into the phenomenon of mistreatment during childbirth in Uttar Pradesh, India. The issue of mistreatment is common and may be underreported by women. Newer and better trained providers may be less likely to mistreat women, suggesting improvements in quality of care training over time. Training modules for services providers can also focus on interpersonal skills and sensitize providers to mistreatment of women during childbirth and its potential implications on outcomes. These findings also suggest associations between provider age with mistreatment suggesting younger providers are less likely to mistreat women, perhaps because younger women have a greater awareness of these issues. A review of pre-service education and inclusion of modules on mistreatment in the curriculum can also prove effective in reducing incidents of mistreatment in the longer run. The issue of under reporting by women also highlights the need for focused interventions directed towards women and their families so that they appreciate respectful maternal care, demand better quality care, and report poor quality of care experiences when they occur. While the study highlights discordance between self-report and observed measures, the findings of the current study do not provide sufficient evidence to justify what issues are more validly captured based on observations vis-à-vis self-report. This is an important area for future research to consider. For now, we recommend inclusion of both types of measurement, observation and patient self-report, and perhaps provider reports as well, which we were not able to include in the current study. Capturing both self-report and observed mistreatment will help provide a better picture of what is going on and how to improve it, as biases in expectations of providers or the childbirth experience can affect reporting on either side. Because this is an emerging area of interest in the field of maternal and reproductive health, there is much work to be done to understand the optimal methods of measuring mistreatment, as well as the factors that contributing to its occurrence. This paper offers insights into both issues, while laying the foundation for the future research necessary to begin the important work of preventing this phenomenon.

## Abbreviations

ANM: Auxiliary nurse midwife
AOR: Adjusted odds ratio
CI: Confidence interval
DHS: Demographic Health Survey
DOD: Direct observation of delivery
GoUP: Government of Uttar Pradesh
HMSC: Health ministry screening committee
ICMR: Indian Council for Medical Research
IRB: Institutional review board
MLE: Measurement learning and Evaluation
MOIC: Medical Officer in Charge
NFHS: National Family Health Survey
NHM: National Health Mission
OBC: Other Backward Classes
PHS-ERB: Public Health Service- Ethical Review Board
SBA: Skilled Birth Attendant
SC: Scheduled Caste
ST: Scheduled Tribe
UP-TSU: Uttar Pradesh Technical Support Unit.
